# Causal association of sarcopenia with hepatocellular carcinoma risk in European population: a Mendelian randomization study

**DOI:** 10.3389/fnut.2024.1292834

**Published:** 2024-05-27

**Authors:** Jiali Cao, Yumei Huang, Mengpei Zhu, Ziwen Wang, Ze Jin, Zhifan Xiong

**Affiliations:** ^1^Department of Gastroenterology, Liyuan Hospital, Tongji Medical College of Huazhong University of Science and Technology, Wuhan, China; ^2^Institute of Geriatric Medicine, Liyuan Hospital, Tongji Medical College of Huazhong University of Science and Technology, Wuhan, China

**Keywords:** sarcopenia, C-reactive protein, hepatocellular carcinoma, Mendelian randomization, appendicular lean mass, grip strength

## Abstract

**Background:**

The causal association of sarcopenia with the incidence risk of hepatocellular carcinoma (HCC) in the European population, and the potential mediating role of C-reactive protein (CRP), remains unclear. This study employed a bidirectional two-sample, two-step Mendelian randomization (MR) analysis to investigate the causality and identify the mediator.

**Methods:**

Summary statistics for HCC, CRP, and sarcopenia-related traits, including appendicular lean mass (ALM), hand grip strength (HGS), and walking pace (WP), were acquired from publicly available databases. We conducted bidirectional MR and Steiger tests of directionality to check the presence of reverse causality. Additionally, a two-step MR analysis was used to assess the mediating effect of CRP in the causality between sarcopenia and HCC. Tests for heterogeneity and horizontal pleiotropy were performed.

**Results:**

As ALM increases, the risk of HCC occurrence decreases [odds ratio (OR), 95% confidence interval (CI): 0.703, 0.524–0.943; *P* = 0.019]. And, genetically predicted low-HGS (OR, 95%CI: 2.287, 1.013–5.164; *P* = 0.047) was associated with an increased incidence risk of HCC, with no reverse causality. However, we found no evidence supporting a causality between WP and HCC. CRP was identified as the mediator of the causal effect of ALM and low-HGS on HCC, with corresponding mediating effects of 9.1% and 7.4%.

**Conclusions:**

This MR study effectively demonstrates that lower ALM and low-HGS are linked to an elevated risk of HCC within the European population, and the causality was not bidirectional. Furthermore, CRP serves as a mediator in the associations. These findings may help mitigate HCC risk among individuals with sarcopenia.

## 1 Introduction

Liver cancer is one of the most prevalent malignancies globally, with hepatocellular carcinoma (HCC) accounting for more than 80% of liver cancers (1). Although the global fatality rate of HCC has exhibited a modest decline over the last decade, it remains alarmingly high (2). It is predicted that the annual incidence of HCC will exceed one million cases by 2025 (3).

Currently, the only widely accepted definition of sarcopenia originates from the European Working Group on Sarcopenia in Older People (EWGSOP) and updated to EWGSOP2 in 2019. In both clinical practice and scientific research, EWGSOP2 delineates sarcopenia as the concurrent presence of low muscle strength and low muscle mass or quality for diagnosis. Moreover, the severity of sarcopenia is ascertained if the patient exhibits concomitant low physical performance (4, 5). Sarcopenia, an age-related degenerative disorder affecting skeletal muscle quality and function, profoundly compromises mobility, nutritional status, and independence (4). The known risk factors associated with sarcopenia can be classified into five categories. Firstly, intrinsic factors encompass age, sex hormone deficiency, comorbidities (e.g., diabetes), and genetic factors. Secondly, body composition, including significant weight loss and sarcopenic obesity (6). Thirdly, lifestyle habits, comprising prolonged immobilization and low physical activity (7). Fourthly, dietary aspects, include low protein intake (8) and vitamin D deficiency (9). Lastly, pharmacological interventions, notably the utilization of ACEI or steroids (6).

At present, many observational studies have proved that sarcopenia is closely related to poor survival outcomes and prognosis of HCC subsequent to various therapeutic modalities, including hepatectomy (10–12), radiofrequency ablation therapy (13, 14), liver transplantation (15), trans-arterial chemoembolization (16), and systemic therapies (17–19). Nevertheless, a paucity of studies have investigated the association of sarcopenia with the incidence risk of HCC. Currently, merely two cohort studies based on Asian populations have proffered evidence that sarcopenia is an independent risk factor for HCC (20, 21). There has been a lack of research on the nexus between sarcopenia and HCC within the European population so far. Given the obvious difference in the impact of sarcopenia on HCC survival outcomes between Eastern and Western populations (11), it is necessary and important to investigate the causal influence of sarcopenia on the risk of HCC within the Western population.

C-reactive protein (CRP), a plasma protein, demonstrates escalated circulatory levels during inflammation and after tissue damage. A pioneering study as early as 1989 showed that serum CRP could be used as a diagnostic biomarker for HCC (22). Recently, many studies have indicated that the level of CRP holds predictive value concerning the prognosis and therapeutic effect of HCC (23–25). Meanwhile, some studies have shown that sarcopenia is closely related to CRP (26, 27). According to the above, CRP might be associated with both sarcopenia and HCC, suggesting that CRP could potentially serve as a risk factor for sarcopenia in people with HCC. Consequently, the exploration of the mediating role of CRP in the relationship between sarcopenia and HCC could enhance the comprehension of the pathogenesis of sarcopenia to HCC and provide potential targets for reducing sarcopenia-related HCC risk. So far, however, few researches have studied the mediating pathway from sarcopenia to HCC.

Mendelian randomization (MR) analysis effectively evaluates the causality between exposures and outcomes by utilizing genetic variations. Compared with traditional observational studies, MR can prevent the interference of unmeasured confounders. To the best of our knowledge, the application of the MR methodology to elucidate the causal impact of sarcopenia on HCC risk within Western populations remains an unexplored domain. Thus, we performed an MR study to investigate the genetic association between sarcopenia and HCC and to evaluate the extent of CRP's mediating role in these associations. This research aims to bridge the gap in the current investigation of the relationship between sarcopenia and HCC within Western populations utilizing MR analysis. Our study may offer a novel perspective for identifying individuals at high risk of HCC and preventing sarcopenia-related HCC.

## 2 Materials and methods

### 2.1 Study design

**Figure 1:**
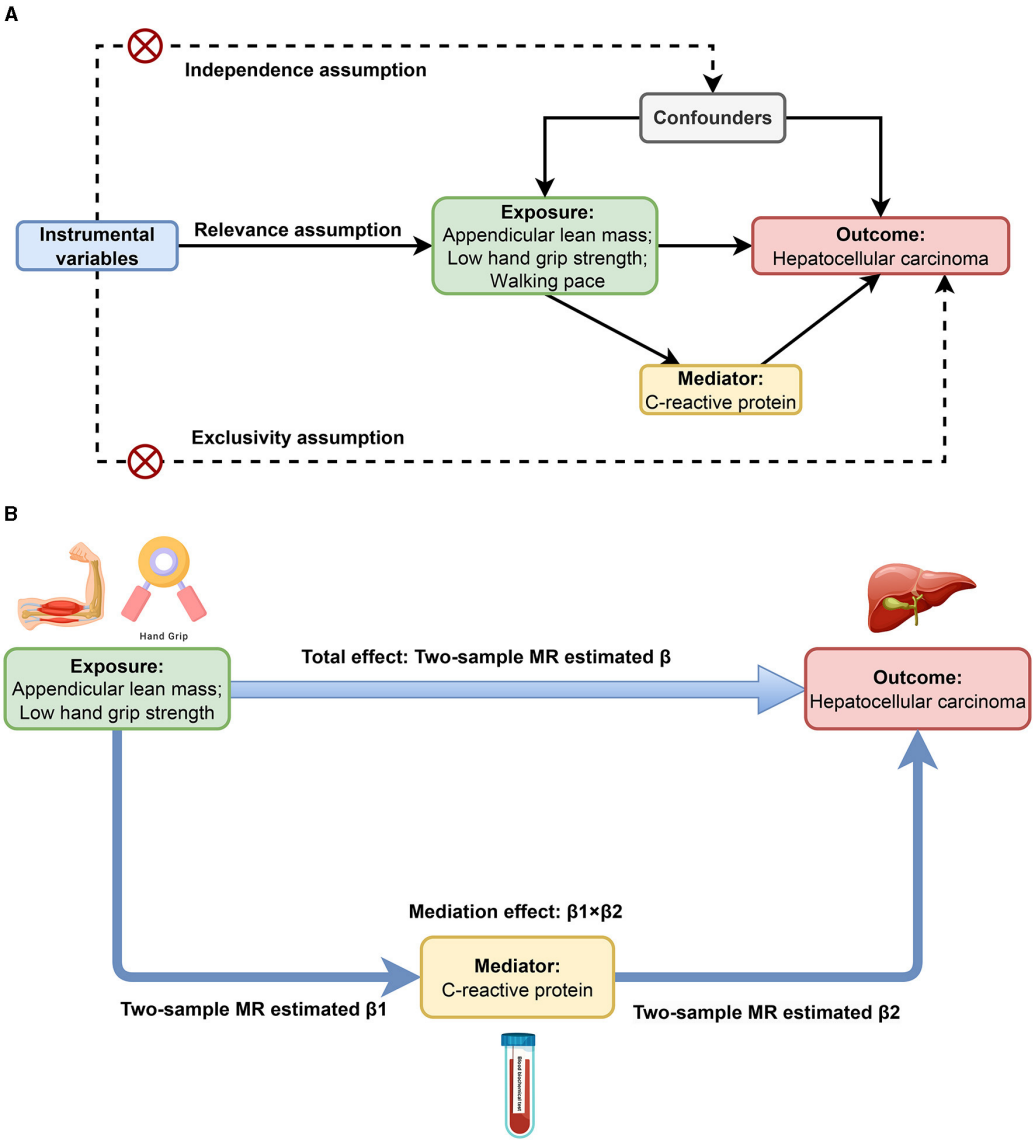
Study design overview. **(A)** Three critical assumptions of MR analysis. **(B)** Two-step MR framework.

This MR study mainly consists of two stages of analysis. Initially, we conducted bidirectional MR analyses to identify and estimate the causal associations of sarcopenia-related traits with HCC. Subsequently, we evaluated CRP's mediating role in the causal links between sarcopenia-related traits and HCC. As shown in Figure 1A, the MR analysis has three critical assumptions: (1) the relevance assumption: the instrumental variables (IVs) are associated with the exposures; (2) the independence assumption: the IVs cannot be connected to any known confounders; and (3) the exclusivity assumption: the IVs do not affect the outcome except through the exposures (28). This MR study employed publicly available GWAS datasets. Within the original GWAS, all study participants provided written informed consent.

### 2.2 GWAS data sources

EWGSOP2 delineates sarcopenia as the concurrent presence of low muscle strength and low muscle mass or quality for diagnosis. Moreover, low physical performance is used to ascertain the severity of sarcopenia. Muscle strength is assessed through hand grip strength (HGS) measurement or chair stand tests. According to the EWGSOP, HGS < 27 kg (male) or < 16 kg (female), or chair stand test >15 seconds for five rises, indicates low muscle strength. Evaluation of muscle mass or quality typically involves dual-energy X-ray absorptiometry, CT, MRI, or magnetic resonance spectroscopy. Appendicular lean mass (ALM) < 20 kg (male), < 15 kg (female) is defined as low muscle quantity. Physical performance is gauged by walking pace (WP), the timed-up-and-go (TUG) test, or completion of a 400-meter walk. And, WP ≤ 0.8 m/s, or TUG ≥20 s, or incomplete 400 m walking or completion exceeding 6 min, denotes low physical performance (5). Genetic instruments for ALM were derived from the UK Biobank, involving 450,243 individuals of European descent (29). Genetic instruments for low-HGS were derived from GWAS data encompassing 135,468 European individuals sourced from the Musculoskeletal Knowledge Portal (30). Genetic instruments for WP (*n* = 459,915) sourced from the MRC Integrative Epidemiology Unit (MRC-IEU). CRP in the blood is a biomarker of inflammation and is measured by blood biochemical tests. GWAS data for CRP were derived from the GWAS by UK Biobank based on 343,524 European individuals (31). To mitigate sample overlap with the exposure GWASs, summary statistics for HCC were from the FinnGen DF9 dataset (453 cases and 287,137 controls) (32). Supplementary Table S1 lists the detailed information of each GWAS summary data in this study.

### 2.3 Genetic instrumental variable selection

The genetic IVs selection adhered to the following principles: Firstly, we incorporated SNPs demonstrating significant genome-wide associations with the exposure variables, adhering to stringent thresholds (*P* < 5 × 10^−8^; *P* < 5 × 10^−6^ for more SNP). Secondly, SNPs independent of each other (linkage disequilibrium [LD] r2 < 0.001 within 10,000 kb) were deemed suitable for inclusion (33). Thirdly, MR-PRESSO was used to remove outliers (34). Finally, the F-statistics were defined as F = $\beta_{\text{exposure}}^{2}$/SE${\;}_{\text{exposure}}^{2}$ to quantify the potency of each SNP, and SNPs with F value < 10 were considered as weak instruments and excluded (35). Detailed IVs data included in this research are shown in Supplementary Tables S2–S9.

### 2.4 Mendelian randomization

We conducted a bidirectional two-sample MR analysis to explore the relationship between sarcopenia-related traits and HCC, and the estimate was the total effect (β). As shown in Figure 1B, we further performed a two-step MR to investigate whether CRP mediates the causal associations between sarcopenia-related traits and HCC. The first step involved the application of a two-step MR approach to estimate the causal effect of each sarcopenia-related trait on CRP, and the estimate was denoted as β1. Meanwhile, we used Steiger test of directionality to examine whether there was a reverse causal relationship between CRP and each sarcopenia-related trait. In the second step, we estimated the causal impact of CRP on HCC employing two-sample MR in conjunction with multivariate MR (MVMR), adjusting for each sarcopenia-related trait. And the two-sample MR estimate was recorded as β2. Reverse MR was used to ensure no reverse causality between HCC and CRP. The mediation effect was calculated as β1 × β2, and the ratio of the mediation effect to the total effect was quantified as R = (β1 × β2)/β (36).

In two-sample MR, we primarily utilized the random effects inverse variance weighting (IVW) analysis method to ascertain the causal relationship between exposures and outcomes. At the same time, MR–Egger (37), weighted median (38), simple mode, and weighted mode (39) were used as supplementary analysis methods. IVW was the primary method of MVMR analysis, while MR–Egger was the auxiliary method.

### 2.5 Sensitivity analysis

For sensitivity analysis, we implemented three approaches: heterogeneity test, horizontal pleiotropy test, and “leave-one-out” method. Heterogeneity was assessed using Cochrane's Q-test, where a Q *p* value < 0.05 was considered indicative of heterogeneity (40). In the presence of heterogeneity, we applied the IVW method with random effects for our study. To identify the presence of horizontal pleiotropy, we scrutinized the statistical significance of the MR–Egger intercept term. In addition, we employed the global test within MR-PRESSO to examine the presence of pleiotropy in our study (34, 41). To evaluate the influence of a single SNP on the causal association, we conducted a “leave-one-out” analysis to eliminate each SNP in turn.

### 2.6 Statistical analysis

The two-sample MR analysis was carried out utilizing R (version 4.2.0) and the package “TwoSampleMR.” MVMR was performed using the “TwoSampleMR,” “MVMR,” and “Mendelian Randomization” R packages. In our MR analysis, SNPs with an *F* value < 10 were excluded from the study. IVW was the primary analysis to estimate the associations and statistical significance was indicated by a threshold of *P* < 0.05, signifying the presence of a significant causal relationship. We presented MR estimates as odds ratios (OR) with 95% confidence intervals (CI) for binary outcomes (HCC and low-HGS) and β coefficients with standard error (SE) for continuous outcomes (ALM, WP, and CRP). The calculation of standard errors and CIs was performed using the delta method (42). In order to evaluate the robustness of IVW results, we further performed sensitivity analysis.

## 3 Results

### 3.1 Causal effects of sarcopenia-related traits on HCC

**Figure 2:**
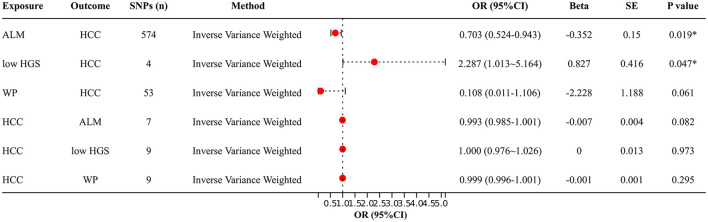
Two-sample MR analysis results. IVW method was used to evaluate the causal relationship between ALM, low-HGS, WP and HCC. OR > 1 signifies an increased risk associated with the exposure indicator, while OR < 1 indicates a decreased risk of the outcome. ALM, appendicular lean mass; low-HGS, low hand grip strength; WP, walking pace; HCC, hepatocellular carcinoma. The symbol ^*^ indicates statistical significance.

**Figure 3:**
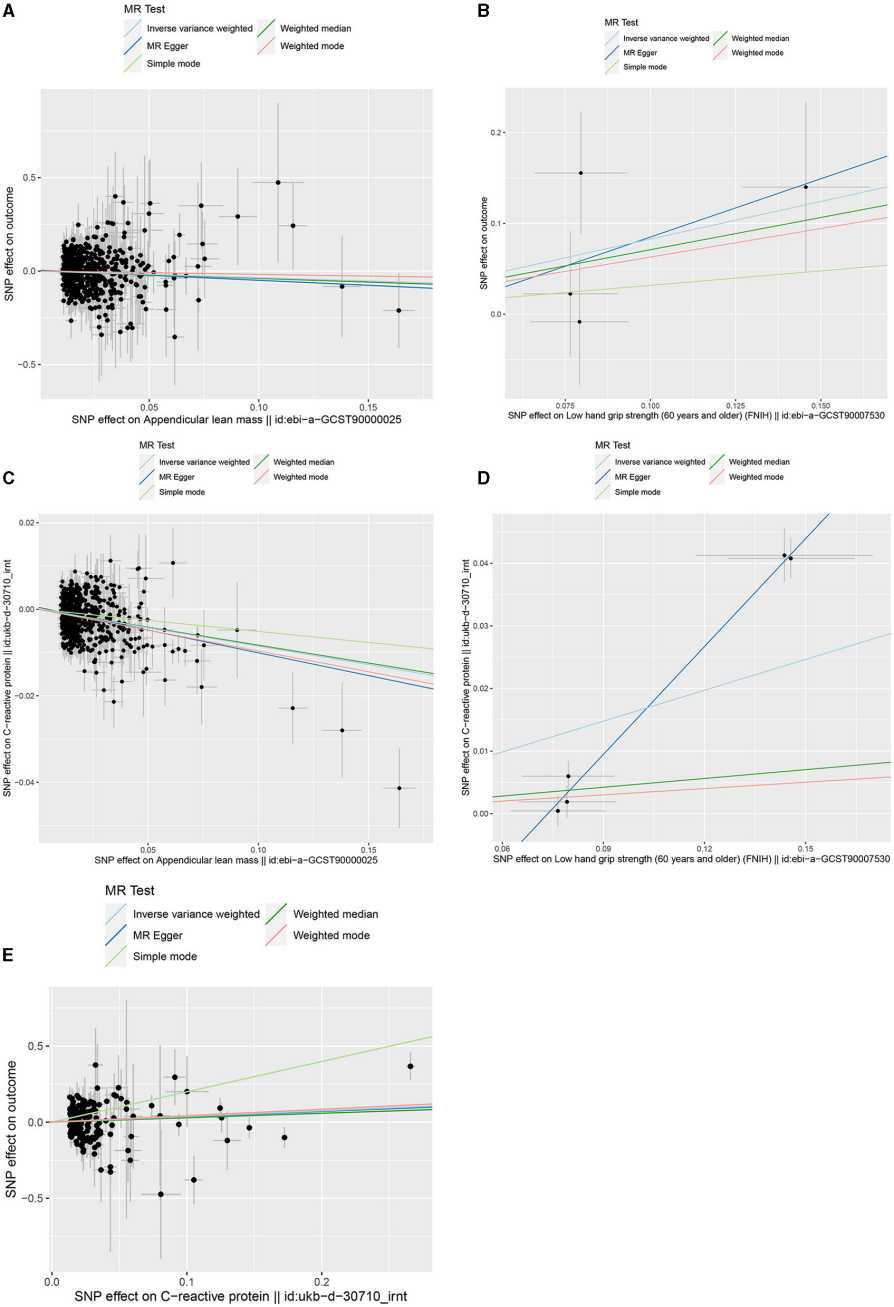
Scatter plots of genetic correlation by different MR analysis methods. **(A)** Scatter plot of genetic correlation between ALM and HCC. **(B)** Scatter plot of genetic correlation between low-HGS and HCC. **(C)** Scatter plot of genetic correlation between ALM and CRP. **(D)** Scatter plot of genetic correlation between low-HGS and CRP. **(E)** Scatter plot of genetic correlation between CRP and HCC.

Figure 2 shows the bidirectional MR results concerning the causal relationship between sarcopenia-related traits and HCC using the IVW method. The results indicate a negative correlation between ALM and HCC (OR = 0.703, 95% CI: 0.524–0.943, *P* = 0.019), as well as a positive correlation between low-HGS and HCC (OR = 2.287, CI: 1.013–5.164, *P* = 0.047). Notably, the scatter charts portraying the trend of fitting results reveal that as ALM increases, the risk of HCC decreases, whereas low-HGS is associated with an elevated risk of HCC (Figures 3A, B). However, according to the two-sample MR results, there was no significant causal relationship between WP and HCC. In reverse MR analysis, no causal effect of HCC on ALM, low-HGS or WP was observed, implying that the genetic predisposition to HCC did not exert an impact on any sarcopenia-related traits (Figure 2). The complete MR results using other methods, including MR Egger, weighted median, simple mode and weighted mode, are presented in Supplementary Table S10.

### 3.2 Causal effects of ALM or low-HGS on CRP

Based on the aforementioned analysis, no causal relationship was observed between WP and HCC. Therefore, in the subsequent two-step MR analysis, our focus shifted exclusively to examining the mediating role of CRP in the causal associations of ALM and low-HGS with HCC. As detailed in Table 1, there was a significant negative causal relationship between ALM and CRP (β_IVW_ = −0.085, 95%CI: −0.1 to −0.07, *P* = 2.59 × 10^−29^), while a significant positive causal relationship between low-HGS and CRP (β_IVW_ = 0.164, 95%CI: 0.042 to 0.286, *P* = 0.008). To validate the causal direction, we conducted a Steiger test of directionality, affirming the correctness of the causal direction (*P* < 0.05) (Table 1). This result implies that CRP does not exert a reverse causal effect on ALM or low-HGS. As shown in Figures 3C, D, the scatter plots showed the trend of fitting results.

**Table 1:** Two-sample MR analysis of the causal relationships between ALM/low-HGS and HCC.

	**SNPs (n)**	**Method**	**Beta (95%CI)**	**SE**	***P* value**	**Correct causal direction**	**Steiger Pval**
ALM on CRP	592	MR Egger	−0.105 (−0.14 to −0.071)	0.018	5.62 × 10^−9*^	TRUE	0
		Weighted median	−0.083 (−0.103 to −0.062)	0.011	2.90 × 10^−15*^		
		IVW	−0.085 (−0.1 to −0.07)	0.008	2.59 × 10^−29*^		
		Simple mode	−0.051 (−0.13 to 0.028)	0.04	0.205		
		Weighted mode	−0.096 (−0.143 to −0.05)	0.024	5.54 × 10^−5*^		
Low-HGS on CRP	5	MR Egger	0.574 (0.487 to 0.661)	0.044	0.001^*^	TRUE	0.003
		Weighted median	0.047 (0.001 to 0.093)	0.024	0.047^*^		
		IVW	0.164 (0.042 to 0.286)	0.062	0.008^*^		
		Simple mode	0.033 (−0.010 to 0.076)	0.022	0.202		
		Weighted mode	0.033 (−0.006 to 0.073)	0.020	0.168		

ALM, appendicular lean mass; low-HGS, low hand grip strength; CRP, C-reactive protein; SNP, single nucleotide polymorphism; Beta, allele-related effects; CI, confidence interval; SE, standard error; Beta, SE, and *P* value are SNP summary statistics; IVW, Inverse variance weighting. The symbol ^*^ indicates statistical significance.

### 3.3 Causal effect of CRP on HCC

**Figure 4:**
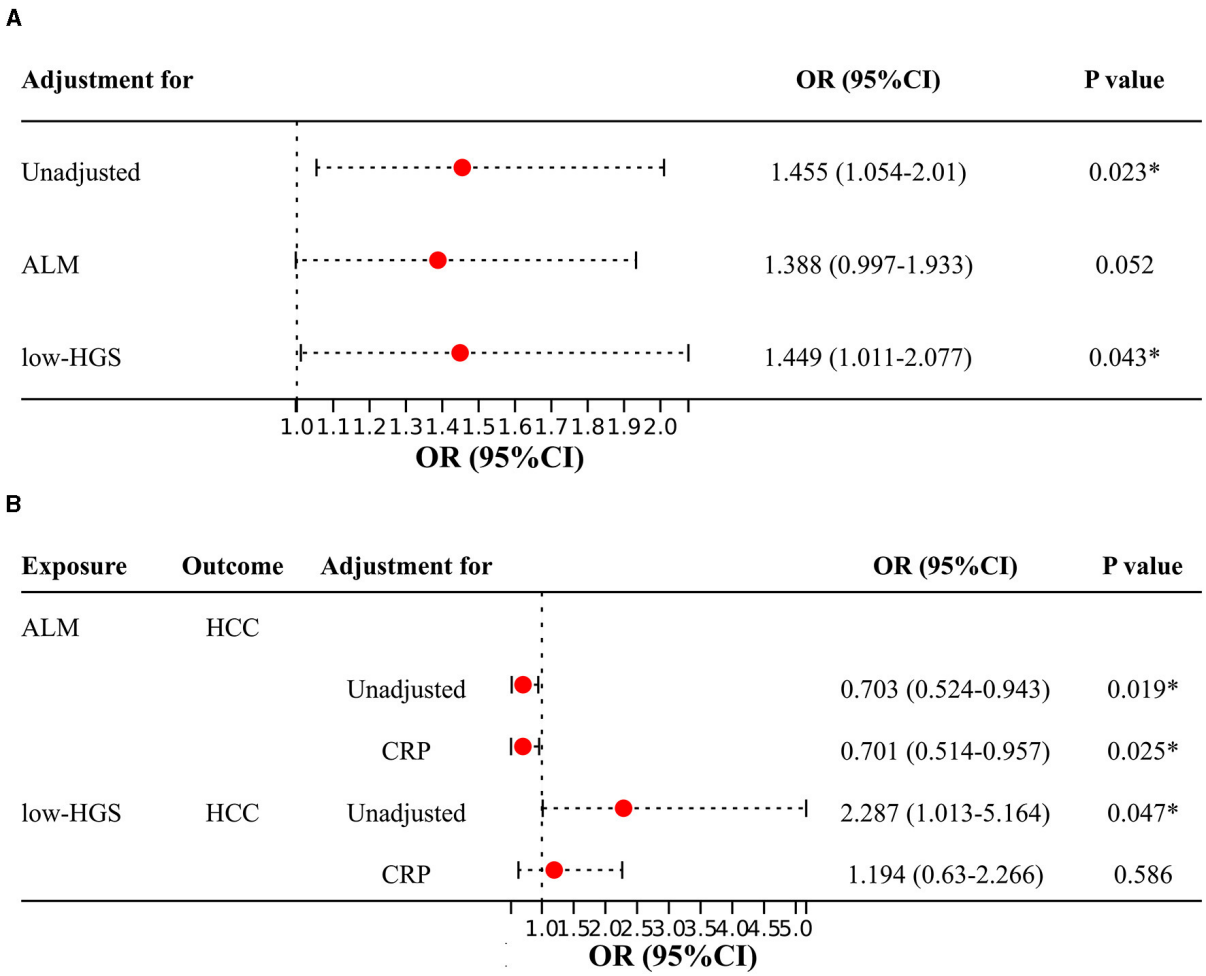
Two-sample MR and MVMR estimates for the causal associations of CRP, ALM and low-HGS with HCC. **(A)** Causal effects of CRP on HCC with adjustment for ALM or low-HGS. **(B)** Causal effects of ALM or low-HGS on HCC with adjustment for CRP. ORs (95% CIs) represent risks for HCC associated with CRP. ALM, appendicular lean mass; low-HGS, low hand grip strength; CRP, C-reactive protein. The symbol ^*^ indicates statistical significance.

In two-sample MR analysis, it was discerned that elevated levels of CRP were significantly linked to an increased risk of HCC (OR = 1.455, 95%CI: 1.054–2.010, *P* = 0.023) (Figure 3E, Supplementary Table S10). In reverse MR analysis, no causal effect of HCC on CRP was observed (*P* = 0.437) (Supplementary Table S10). In MVMR, the causal association of CRP with HCC was diminished with adjustment for ALM, while low-HGS had no significant effect on it (Figure 4A). Detailed MVMR results can be found in Supplementary Table S11.

### 3.4 Mediation effect of CRP in the associations between sarcopenia-related traits and HCC

The above two-step MR analysis results highlight the mediating role of CRP in the causal associations involving ALM or low-HGS with HCC. As shown in Figure 4B, in MVMR, the causal association of ALM with HCC remained relatively unchanged upon adjustment for CRP. In contrast, the causal association of low-HGS with HCC was notably diminished with adjustment for CRP, indicating that this association was largely affected by CRP. Specifically, CRP explained 9.1% (95% CI: 1.23%−16.95%) of the total effect of ALM on HCC (Table 2). The proportion mediated by CRP in the associations between low-HGS and HCC was 7.4% (95% CI: 3.11%−17.98%) (Table 2).

**Table 2:** Mediation effect and mediation proportion by CRP.

**Exposure - Mediator - Outcome**	**β1 (95%CI)**	**β2 (95%CI)**	**β (95%CI)**	**Mediation effect(95%CI)**	**Mediation proportion (95%CI)**
ALM – CRP - HCC	−0.085 (−0.1 to −0.07)	0.375 (0.052 to 0.698)	−0.352 (−0.645 to −0.059)	−0.032 (−0.06 to 0.004)	9.1% (1.23% to 16.95%)
Low-HGS – CRP - HCC	0.164 (0.042 to 0.286)	0.375 (0.052 to 0.698)	0.827 (0.012 to 1.642)	0.0615 (−0.011 to 0.134)	7.4% (3.11% to 17.98%)

β1, MR estimate of exposure (ALM or low-HGS) on mediator (CRP); β2, MR estimate of mediator (CRP) on outcome (HCC); β, MR estimate of exposure (ALM or low-HGS) on outcome (HCC); Mediation Effect = β1 × β2; Mediation proportion = (β1 × β2)/β; ALM, appendicular lean mass; low-HGS, low hand grip strength; CRP, C-reactive protein; HCC, hepatocellular carcinoma.

### 3.5 Sensitivity analyses

In our two-sample MR analysis, we employed IVW and MR–Egger methods to discern heterogeneity, while MR-PRESSO and MR–Egger analyses were harnessed to detect horizontal pleiotropy. As outlined in Table 3, MR analysis of ALM on CRP had heterogeneity, so IVW with random effects analysis was used in MR analysis. Except for this, there was no heterogeneity in other two-sample MR analyses (*P* > 0.05). Moreover, no evidence of horizontal pleiotropy was detected in any of our two-sample MR analyses (*P* > 0.05). The funnel plots illustrating the heterogeneity test results were presented in Supplementary Figure S1. Furthermore, the “leave-one-out” analysis revealed minimal fluctuations in the error line, indicating the robustness of the MR results (Supplementary Figure S2).

**Table 3:** Sensitivity analysis for two-sample MR and MVMR, including heterogeneity test and horizontal pleiotropy test.

**Exposure**	**Outcome**	**Heterogeneity test**						**Horizontal pleiotropy test**		
		**MR–Egger**			**IVW**			**MR–Egger**		**MR-PRESSO**
		**Cochrane's Q**	**Q_df**	**Q_pval**	**Cochrane's Q**	**Q_df**	**Q_pval**	**Intercept**	**Pval**	**Global test pval**
**Two-sample MR**										
ALM	HCC	564.839	572	0.576	565.204	573	0.584	0.005	0.546	0.571
Low-HGS	HCC	3.176	2	0.204	3.273	3	0.351	−0.043	0.828	0.434
WP	HCC	52.823	51	0.403	53.287	52	0.424	−0.030	0.507	0.445
HCC	ALM	6.144	5	0.292	12.799	5	0.046	−0.012	0.067	0.093
HCC	Low-HGS	4.469	7	0.724	4.474	8	0.812	−0.002	0.942	0.839
HCC	WP	5.226	7	0.632	5.652	8	0.686	0.001	0.535	0.741
ALM	CRP	1,166.411	590	3.36 × 10^−40^	1,169.5	591	2.2 × 10^−40^	0.000	0.212	0.977
Low-HGS	CRP	10.982	3	0.687	12.767	4	0.620	0.003	0.203	0.642
CRP	HCC	200.937	174	0.079	200.978	175	0.087	0.002	0.851	0.06
HCC	CRP	3.367	5	0.644	6.358	6	0.384	−0.006	0.144	0.419
**MVMR**										
ALM and CRP	HCC	541.107	534	0.406	541.256	535	0.417	−0.003	0.701	0.325
Low-HGS and CRP	HCC	178.511	160	0.151	179.844	161	0.147	0.010	0.274	0.065

df, degrees of freedom; IVW, Inverse variance weighting; ALM, appendicular lean mass; low-HGS, low hand grip strength; WP, walking pace; HCC, hepatocellular carcinoma; CRP, C-reactive protein.

In each MVMR analysis, both IVW and MR-Egger methods indicated the absence of heterogeneity, and the MR–Egger and MR-PRESSO methods confirmed the absence of horizontal pleiotropy (Table 3).

## 4 Discussion

Our MR study systematically investigated the causal effects of sarcopenia-related traits, including ALM, low-HGS, and WP, on HCC. Simultaneously, the potential intermediation of CRP within these associations was explored. The results revealed that genetically determined lower ALM and low-HGS were causally associated with increased risk of HCC, but no significant causal relationship was discerned between WP and HCC. And there is no reverse causal relationship between HCC and sarcopenia-related traits. In addition, we assessed the mediating role of CRP in the causal connections between sarcopenia-related traits and HCC. And the mediating influence of CRP was quantified, revealing mediation proportions of 9.1% for the effect of ALM and 7.4% for the effect of low-HGS on HCC. It is noteworthy that the causal link between ALM and HCC remained substantively unchanged upon the adjustment for CRP. However, the causal association of low-HGS with HCC was diminished with CRP adjustment, suggesting this association was largely affected by CRP. Taken collectively, these findings support causal impacts of lower ALM and low-HGS on greater risks of HCC, demonstrating the substantial mediating role of CRP between sarcopenia and HCC. There is no horizontal pleiotropy in MR analyses, and the MR results are robust through “leave-one-out” analysis.

Sarcopenia significantly affected a series of adverse health-related outcomes, especially in patients with tumors and the geriatric population (43). EWGSOP used muscle mass and muscle strength to define sarcopenia, and physical performance was used to assess the severity (5). A previous systematic review and meta-analysis showed that in patients with HCC, skeletal muscle mass loss is associated with increased all-cause mortality and tumor recurrence (44). However, a cohort study of the Dutch population yielded contrasting findings, as no significant correlation emerged between sarcopenia and the overall survival of HCC (11). Such discrepancy in conclusions potentially stems from ethnic heterogeneity, considering the distinct ancestral backgrounds of the objects of these studies. HGS is the most robust indicator of muscle strength. A cohort study of the Japanese population showed that low HGS was a poor prognostic factor for unresectable HCC patients treated with lenvatinib (45). In addition, a retrospective study showed that reduced HGS in patients with chronic liver diseases was an independent adverse predictor of mortality (46). WP serves as an indicator of physical performance. According to the EWGSOP criteria, walking speed < 0.8 m/s is defined as slow WP (5). At present, investigations about the relationship between WP and HCC are sparse, with limited available data. A solitary study revealed that there was no significant difference in the prevalence of complications, delirium or falls after HCC treatment between the slow WP group and the normal group (47). Viral hepatitis and alcoholic hepatitis are significant causes of HCC, with sarcopenia being a very common comorbidity (48). The prevalence of sarcopenia is higher and prognosis poorer among patients with chronic viral hepatitis (49). Alcoholic hepatitis may exacerbate sarcopenia due to liver damage and malnutrition (50). Additionally, sarcopenia is believed to facilitate the progression of chronic hepatitis and the transformation into HCC (50). Hence, hepatitis and concurrent sarcopenia may jointly contribute to the occurrence and progression of HCC.

Our MR results unveiled a fresh perspective that genetically predicted lower muscle mass and muscle strength were strong risk factors for HCC within European population. Besides, we observed a more independent effect of ALM compared to HGS on HCC with adjustment for CRP. However, the MR investigation does not reveal a significant relationship between WP and HCC. Given that WP is not a necessary factor in the diagnostic criteria of sarcopenia according to the definition of EWGSOP, based on our MR results on the relationship between ALM and HGS and HCC, we can still conclude that there is a causal relationship between sarcopenia and HCC risk.

Notably, our study has shown the mediating role played by CRP and has quantified the proportions mediated by CRP between sarcopenia-related traits and HCC. CRP, an acute-phase reactant, exhibits its production under the regulation of proinflammatory cytokines, as delineated in the extant literature (51). Our MR results suggested that higher serum CRP levels increased the risk of HCC. This result harmoniously resonates with prior researches, wherein serum CRP has been extolled as a diagnostic marker for HCC and an independent marker for predicting poor prognosis and early recurrence (22, 52). A possible explanation for the effect of CRP on HCC is that prolonged inflammation leads to the infiltration of immunocytes into the liver to facilitate tissue remodeling, and additional investigations are necessary to clarify precise molecular mechanisms. Furthermore, our MR analysis unveiled a causal relationship between higher ALM and lower CRP, which is congruent with previous evidence that lean mass has a favorable effect on inflammation (53). Besides, a meta-analysis showed that HGS is negatively correlated with CRP (27), which supported our MR results. Subsidiary to this, our study identified relatively higher mediation proportions by CRP between ALM and HCC compared with low-HGS and HCC (ALM: 9.1%, low-HGS: 7.4%). This may be due to the endocrine function of skeletal muscle that affects systemic metabolism and inflammation, resulting in a more direct association of CRP with ALM than with HGS.

To the best of our knowledge, this study provided reliable causal evidence for the adverse impact of sarcopenia-related traits on HCC risk in European population for the first time, and elucidated a potential CRP-mediated pathway between sarcopenia and HCC. In a word, this study highlights the importance of monitoring muscle mass, strength, and serum CRP levels in individuals with sarcopenia, emphasizing their critical role in reducing the prevalence of HCC and diminishing the related disease burden.

This study boasts several strengths. Primarily, it offered a novel perspective on the causal associations and potential mediation effect between sarcopenia-related traits and HCC within the European population, as ascertained through the MR analysis. Furthermore, the deployment of bidirectional MR analysis and Steiger tests of directionality, has effectively mitigated the biases stemming from reverse causality. Moreover, comprehensive sensitivity analysis and “leave-one-out” analysis ensured the robustness of the results. These rigorous evaluations collectively buttress the robustness and reliability of our results, adding a layer of assurance to our conclusions.

Limitations warrant consideration. Firstly, despite the implementation of multiple sensitivity analyses, our study cannot entirely eliminate the potential interference of confounders, and thus we urge cautious interpretation of the causal associations elucidated herein. Secondly, the absence of individual-level data hindered our capacity to investigate the relationship between sarcopenia-related traits and HCC via subgroup analysis. Subsequent analyses are thus warranted to probe these associations stratified by age and sex. Thirdly, it is essential to emphasize that our study primarily relies on GWAS data from individuals of European descent. Consequently, circumspection is advised when extending our findings to other ethnic groups. Lastly, constraints imposed by the current GWAS database hindered the identification of more optimal mediators. These limitations include factors such as gene coverage in the database, measurement methods of phenotypes, and sample sizes. In the future, it will be necessary to analyze and study in broader GWAS datasets to explore more prominent mediators and to explore the potential mechanisms between sarcopenia and HCC.

In conclusion, this study shed light on the causal associations of lower ALM and low-HGS with increased HCC risk and the mediating role of CRP in the association pathways within the European population. Intervention of these influential factors may offer a promising avenue for preventive measures against HCC in individuals with sarcopenia.

## Data availability statement

The original contributions presented in the study are included in the article/Supplementary material, further inquiries can be directed to the corresponding author.

## Author contributions

JC: Conceptualization, Methodology, Writing—original draft. YH: Data curation, Writing—review & editing. MZ: Writing—review & editing, Formal analysis. ZW: Writing—review & editing, Investigation, Validation. ZJ: Writing—review & editing, Software, Visualization. ZX: Writing—review & editing, Funding acquisition, Resources, Supervision.
