# Supplementary material for: Causal association of sarcopenia with hepatocellular carcinoma risk in European population: a Mendelian randomization study
Source: Front Nutr. 2024 May 27;11:1292834. doi: 10.3389/fnut.2024.1292834 (PMC11163102; doi:10.3389/fnut.2024.1292834)
Supplement: Supplementary file 2 [file Image_1.pdf]

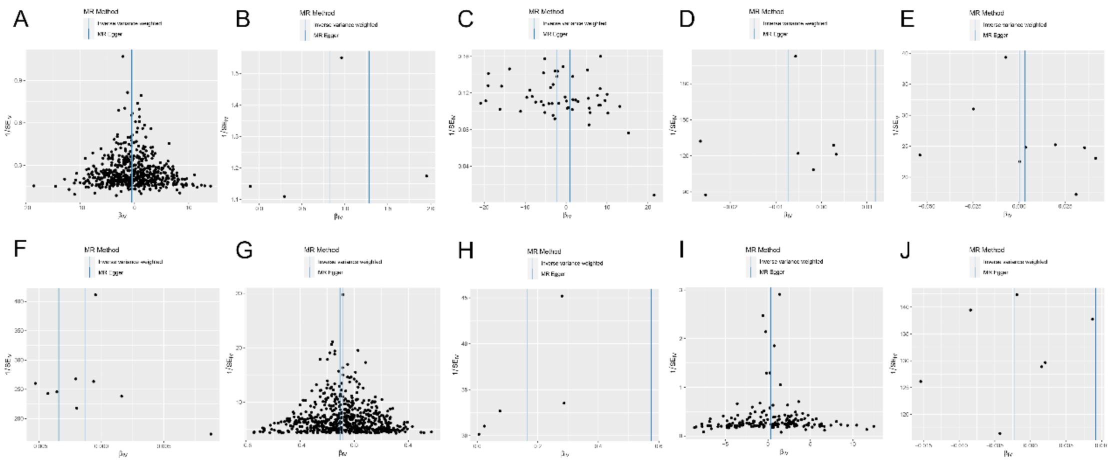

Figure S1 Funnel plots for heterogeneity test. (A) ALM on HCC; (B) low-HGS on HCC; (C) WP on HCC; (D) HCC on ALM; (E) HCC on low-HGS; (F) HCC on WP; (G) ALM on HCC; (H) low-HGS on CRP; (I) CRP on HCC; (J) HCC on CRP. ALM, appendicular lean mass; HCC, hepatocellular carcinoma; low-HGS, low hand grip strength; WP, walking pace; CRP, C-reactive protein.

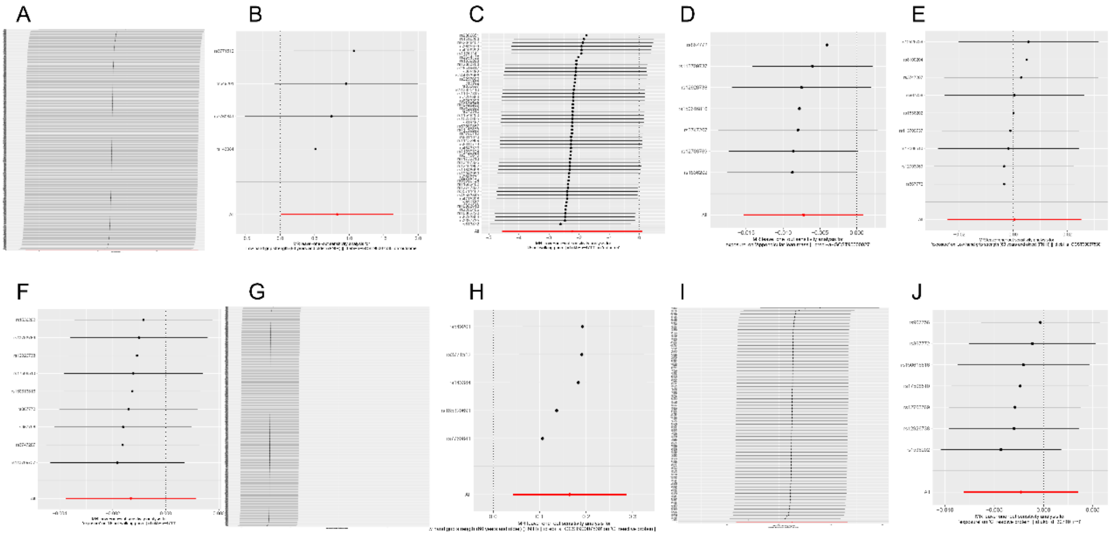

Figure S2 “Leave-one-out” results of genetic correlation. (A) ALM on HCC; (B) low-HGS on HCC; (C) WP on HCC; (D) HCC on ALM; (E) HCC on low-HGS; (F) HCC on WP; (G) ALM on HCC; (H) low-HGS on CRP; (I) CRP on HCC; (J) HCC on CRP. ALM, appendicular lean mass; HCC, hepatocellular carcinoma; low-HGS, low hand grip strength; WP, walking pace; CRP, C-reactive protein.
